# Intraoperative Flow Cytometry for the Characterization of Gynecological Malignancies

**DOI:** 10.3390/biology11091339

**Published:** 2022-09-11

**Authors:** Zoi Anastasiadi, Stefania Mantziou, Christos Akrivis, Minas Paschopoulos, Eufemia Balasi, Georgios D. Lianos, George A. Alexiou, Michail Mitsis, George Vartholomatos, Georgios S. Markopoulos

**Affiliations:** 1 Department of Obstetrics and Gynecology, ‘G. Chatzikosta’ General Hospital, 45001 Ioannina, Greece; 2 Haematology Laboratory-Unit of Molecular Biology, University Hospital of Ioannina, 45500 Ioannina, Greece; 3 Department of Obstetrics and Gynecology, University Hospital of Ioannina, 45500 Ioannina, Greece; 4 Pathology Department, ‘G. Chatzikosta’ General Hospital, 45001 Ioannina, Greece; 5 Department of Surgery, University Hospital of Ioannina, 45500 Ioannina, Greece; 6 Department of Neurosurgery, University Hospital of Ioannina, 45500 Ioannina, Greece; 7 Neurosurgical Institute, Faculty of Medicine, University of Ioannina, 45110 Ioannina, Greece

**Keywords:** cancer, surgical treatment, flow cytometry, gynecological malignancies, surgical oncology

## Abstract

**Simple Summary:**

Aneuploidy and high proliferative potential are distinct features of neoplastic cells. Based on the established role of intraoperative flow cytometry in various types of cancer, the aim of the present study was to investigate its role in cancer cell identification during surgery for gynecological malignancies. The analysis time was 5–6 min per sample. A large percentage of tumors were characterized as aneuploid, while all tumor samples had a significantly high proliferation. Flow cytometry was performed in accordance with pathological evaluation, and the method had high sensitivity and specificity. Our results verify the value of intraoperative flow cytometry in gynecological malignancies, and warrant further investigation in multicenter studies.

**Abstract:**

Cell-cycle analysis has shown the presence of aneuploidy to be associated with poor prognosis. We developed an innovative rapid cell-cycle analysis protocol (the Ioannina protocol) that permitted the intraoperative identification of neoplastic cells in a plethora of malignancies. Herein, we aimed to investigate the potential role of cell-cycle analysis in the intraoperative characterization of gynecological malignancies. Women who underwent surgery for gynecological malignancies in our institution over a three-year period were included in this study. Permanent section pathology evaluation was used as the gold standard for malignancy evaluation. Total accordance was observed between flow cytometry and pathology evaluation. In total, 21 aneuploid cancers were detected following DNA index calculation. Of these, 20 were hyperploid and 1 was hypoploid. In addition, tumor samples were characterized by a significantly lower percentage of cells in G0/G1, as well as an induced tumor index. The response time for flow cytometry to obtain results was 5–6 min per sample. It seems that flow cytometry analyses for intraoperative tumor evaluation can be safely expanded to gynecological malignancies. This is a novel practical approach that has been proven valuable in several tumor types to date, and also seems to be reliable for gynecological malignancies. Intraoperative flow cytometry is expected to be crucial in decisions of lymph node dissection in endometrial cancers, due to its rapid response regarding the tumor invasion of part or all of the myometrial thickness. In this way, the surgeon can quickly modify the plane of dissection. Our results warrant the further investigation of applying iFC in larger, multicenter studies.

## 1. Introduction

Cancer is the second leading cause of human mortality worldwide, recording 10 million cancer deaths in the last year, while future estimations remain dismal; cancer-related incidence and mortality is projected to exceed 28 million new cases and, ominously, 16 million deaths by 2040 [1]. From this perspective, gynecological malignancies represent a major health issue, affecting many organs across the female reproductive tract—mainly the endometrium, cervix, ovaries, and vulva [2,3]—accounting for 1,335,453 new cases and approximately 646,453 deaths in the year 2020 [4]. One distinctive feature of cancer is the constant creation of abnormal cells that defy contact inhibition, invade adjoining tissues, and cause metastasis—the primary cause of death from cancer.

Endometrial cancer is the second most common cause of cancer in women, after breast cancer [5], and can be divided into two main subtypes according to Bokhman’s dualistic model [6]: the most common (type I) tumors are adenocarcinomas (with or without squamous morphological features), often well-differentiated [7], and are reported to be correlated with high serum estrogen levels. The remaining sporadic endometrial tumors—nonendometrioid type II—account for the majority of deaths and recurrences, and are histologically distinguished as uterine papillary serous carcinoma (UPSC) and clear-cell carcinoma [8]. Cervical cancers present as endocervical adenocarcinoma or squamous-cell carcinomas. Vulvar and vaginal cancers, typically diagnosed as squamous-cell carcinomas, account for approximately 4% of malignancies. Uterine sarcomas comprise less than 1% of gynecological malignancies and 2–5% of all uterine malignancies, and include sarcomas (arising in the endometrial stroma), carcinosarcomas (mixed mesodermal sarcomas or Mullerian tumors), and leiomyosarcomas (in the myometrial muscle) [9,10].

Ovarian cancer is reported as one of the most prevalent pathologies in gynecological oncology. This malignancy is characterized as epithelial or non-epithelial [11], and can be categorized into further subgroups: low-grade and high-grade serous cancer, [3] endometrioid, clear-cell, and mucinous ovarian cancer [12]. Many genes have been implicated in pathogenesis, with the most prevalent being the p53, KRAS, PTEN, and MEK and their related signalling pathways. Due to the lack of specific symptoms, and as they exhibit sporadic distribution (only 5–10% of cases are correlated with family history), 70% of ovarian cancer cases are detected in the advanced stages [13], thus having the lowest survival rate among all gynecological cancers [12].

Cancer development and progression are manifested by an interplay between environmental and genetic factors, and each type exhibits distinct morphological and molecular characteristics. Classification of cancer subtype and stage is mainly based on several systems, including clinical, histological, endocrinological, and genetic modifications. The latter involves DNA content measurement, profiling of differentially expressed genes, and identification of a different set of cell surface markers (CDs), which can be used as biomarkers [14,15,16]. This procedure is of critical importance, as it can contribute to the optimization of pharmaceutical approaches and treatment strategies, predict chemoresistance, and assess patients’ prognosis [17]. Previous studies showed that independent prognostic factors such as high mitotic rate, large tumor size, and advanced age were associated with shorter survival [18].

In the fight against cancer, computed tomography (CT) scans and, even more, magnetic resonance imaging (MRI), provide crucial information in determining tumor location and size, and can help delineate tumor boundaries. However, some cancer types—such as cervical tumors—can be indistinguishable from adjacent tissue or appear as a non-specific enlargement due to being isodense [19]. These limitations pose a gap between diagnosis and treatment, as accurate definition of a tumor’s healthy margins has a high prognostic value, and is of paramount importance for assessing the risk of recurrence. Successful surgical resection of a tumor offers complete cancer removal, increases the five-year relative survival rate, and is considered to be of major importance in treatment.

Flow cytometry is among the most powerful cell-specific analytical tools, with several applications in the study of cancer, including cancer cell immunophenotyping, characterization of hematological malignancies, revealing minimal residual disease and metastatic progression, and measuring DNA content and ploidy to study cell cycle progression [20]. Intraoperative flow cytometry (iFC) is a breakthrough technique enabling the cytometric analysis of DNA content/ploidy and cell-cycle distribution of cells acquired during surgical resection of tumors to characterize cancer cells and to evaluate the limits of resection, bridging the aforementioned gap between evaluation and surgery. This procedure has been applied by our team during central nervous tumor surgeries, and its significance has since been confirmed in several additional cancer types, including head-and-neck malignancies, breast cancer, hepatocellular cancer, pancreatic cancer, and colorectal cancer [21,22,23,24,25,26,27,28,29,30]. Intraoperative flow cytometry offers good diagnostic potential with high sensitivity and specificity, while a prognostic role is also suggested. With an analysis time of less than 10 min, iFC is considered to be an accurate next-generation cancer-cell evaluation technique that can be applied intraoperatively [24,27,28,31,32]. The diagnostic and prognostic potential of iFC has been independently verified for central nervous system malignancies by a research team in Tokyo, Japan [33,34,35,36,37].

In the present study, we present a novel approach in defining gynecological tumors’ biology during surgery by applying iFC. Our results indicate the impact of iFC during surgery, and support the utilization of iFC in further multicenter studies.

## 2. Materials and Methods

This study included patients hospitalized in the Department of Obstetrics and Gynecology at the General Hospital of Hatzikosta, over a 3-year period (2018–2021), who underwent surgery for gynecological malignancies. Fifty women (aged 35 to 83 years, with a median age of 64.13 years) were recruited to the study: 36 women with endometrial cancer, 8 women with ovarian cancer, 1 woman with cervical cancer, 2 women with uterine sarcoma, and 3 women with complex endometrial hyperplasia with atypia. During each surgery, a tumor sample with a volume of ~5 mm^2^ was removed with a scalpel from tumor tissue, along with another sample from macroscopic healthy tissue. The sample was divided into two equal pieces; half was sent for flow cytometry analysis and the other half for frozen-section pathology evaluation. Both the pathology and flow cytometry analysis evaluations were blinded. The pathology examination of samples on permanent tissue sections was considered the gold standard, and was performed by an expert pathologist. Informed consent was obtained from every patient. The study was approved by our institutional review board, and was in accordance with the principles of the Declaration of Helsinki.

### 2.1. DNA Content Analysis (Ioannina Protocol)

DNA analysis by the Ioannina protocol was performed immediately after tumor excision [21]. Briefly, tumor samples were thoroughly minced (Medimachine System, BD Bioscience, Franklin Lakes, NJ, USA) for 1 min in phosphate-buffered saline (Ca^2+^- and Mg^2+^-free, with 0.5 mg/mL RNase), and a homogeneous cell suspension was obtained. The suspension was filtered to remove cell aggregates and to obtain single-cell suspensions. Cells were counted using an automated hematology analyzer to a final concentration of 10^6^ cells/mL. Cells were then processed immediately for staining by adding propidium iodide (125 μg/mL) and, after 3 min, flow cytometric analysis was performed. All of the samples were analyzed using a FACSCalibur flow cytometer (BD Bioscience) equipped with two lasers (488 nm, 635 nm) and six parameters (FSC, SSC, FL1–FL4), using CellQuest V3.1 software (BD Bioscience). Normal peripheral blood mononuclear cells obtained from healthy donors using a Ficoll gradient (Ficoll-Paque separation, GE Healthcare, Little Chalfont, Buckinghamshire, UK) were used as the standard for flow cytometer calibration, and to define the diploid G0/G1 peak in the DNA histograms. An average of 5000 events (cell nuclei) counted in each sample were evaluated. A gating strategy based on area/width analysis of propidium iodide fluorescence was utilized to rule out cell doublets from DNA content quantification, as previously described [28]. The total analysis time per sample was 5–6 min.

Following the analysis, two indices were calculated: First, the DNA index, which is the ratio of the geometric mean of the G0/G1 peak of cancer cells to that of normal cells. A cancer with a DNA index of over 1.1 was considered aneuploid/hyperploid, and if the DNA index was below 0.9 it was considered aneuploid/hypoploid. If the DNA index was calculated as 1, the cancer was considered diploid. The tumor index, indicative of cancer cell proliferation, was also calculated as the total sum of the percentage of cells in the S and G2/M phases.

### 2.2. Statistical Analysis

We used the Mann–Whitney U test to compare the G0/G1, S-phase, and mitotic fractions of tumors versus normal cells. Continuous data are expressed as the mean ± standard deviation. Receiver operating characteristic (ROC) curve analysis was used to determine the optimal cutoff values and calculate the sensitivity and specificity of the method. The level of significance was defined as a probability value less than 0.05. Statistical analyses were performed using SPSS V.26 software (IBM) and viewed in GraphPad Prism V 8.4.2 (GraphPad Software, LLC).

## 3. Results

Our study initially included 50 female patients aged 35–81 years (median age: 63 years) who underwent surgery for gynecological malignancies (see Materials and Methods for more details). Cancer cell characterization as well as cancer-to-healthy-tissue margin evaluation is of particular importance in the success of every cancer surgery and patient follow-up, as mentioned elsewhere [38]. We used real-time iFC to determine the proliferative potential of specimen cells in order to designate the status of cancer cells, as a first step towards margin evaluation. To this end, cancer tissue cells from each patient, as well as peripheral mononuclear blood cells (PMBCs) from healthy donors, were used for cytometric analysis.

**Figure 1:**
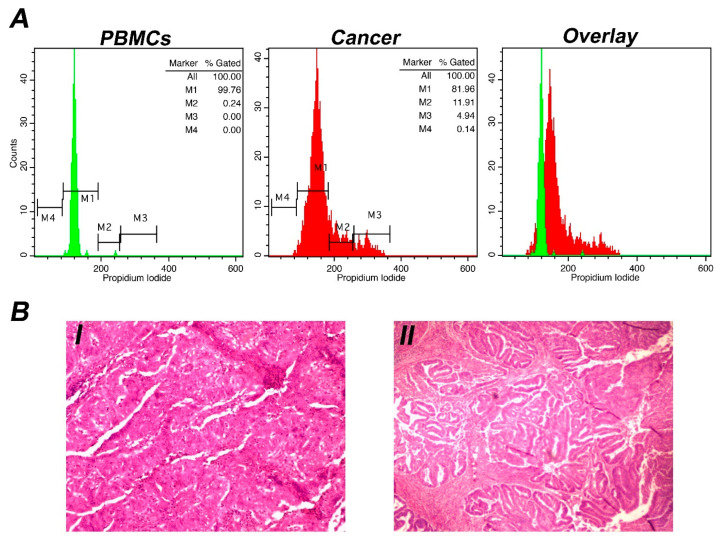
Analysis by iFC and pathology assessment of a representative case of endometrial cancer: (**A**) DNA content and cell-cycle distribution analysis using iFC: Cancer cells acquired during surgery (presented in red histograms) and normal (control) PBMCs (presented in green histograms) were stained with propidium iodide, and the quantity of DNA was analyzed using flow cytometry. Markers M1, M2, and M3 and M4 represent the G1, S and G2/M cell-cycle phases, respectively. Marker M4 represents cells in subG1 (indicative of cell death). The percentage of cells that correspond to each phase is shown in the upper right part of the histogram. The G0/G1 peak from the PBMC sample was used as a reference for DNA index calculation. The presented case is hyperploid, with a DNA index of 1.2 (the G0/G1 peak of cancer cells in red is discernible from that of normal cells in green in the overlaid histogram). Tumor index (i.e., percentage of proliferative cells) was calculated at ~17%. (**B**). Pathology evaluation: Ovarian cancer cells stained with hematoxylin/eosin are presented at 40× (I) and 200× (II) magnification. A moderately differentiated tumor (grade II) is recognized.

Following intraoperative flow cytometry screening, samples from 8/50 patients were characterized as inappropriate for iFC analysis, since they were devoid of cells. The other 42 samples were from 31 women with endometrial cancer, 6 women with ovarian cancer, 1 woman with cervical cancer, 2 women with uterine sarcoma, and 2 women with complex endometrial hyperplasia with atypia, and were included in the final analysis. Individual patients’ characteristics—including age, results of flow cytometry analysis, and the final pathology report—are presented in Table S1. A representative analysis is depicted in Figure 1.

**Figure 2:**
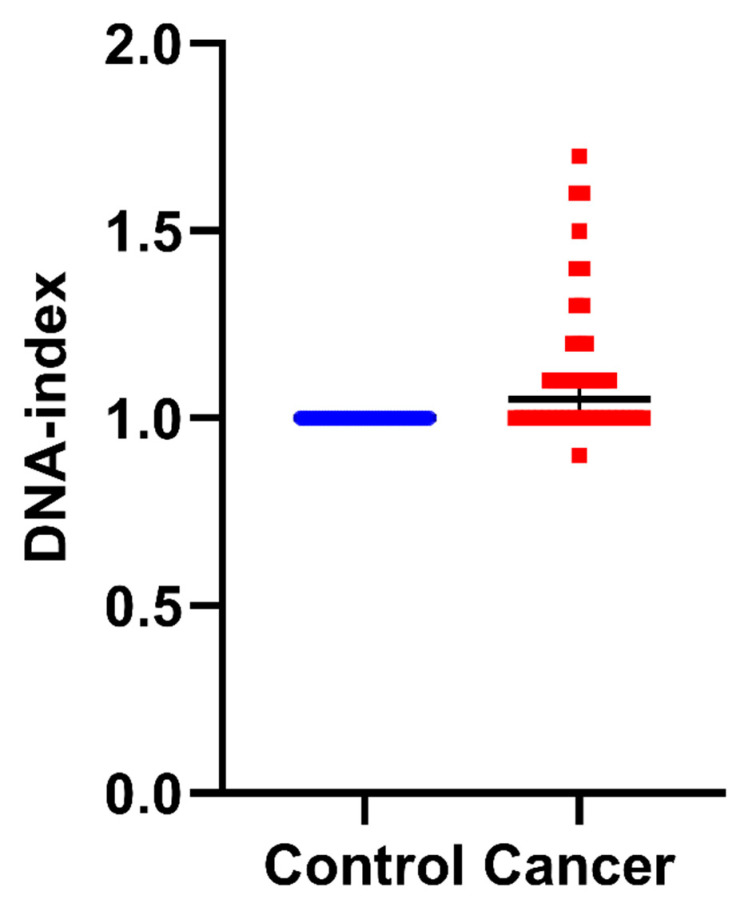
DNA index as an indicator of aneuploidy in normal control samples versus tumor cells: DNA index values were quantified following iFC analysis, as a quotient of the geometric mean of G0/G1 in normal (based on analysis of PBMCs) vs. cancer cells in individual patient samples. DNA index scores of individual samples are presented as blue or red dots, for normal and cancer tissue, respectively. A DNA index of ≠1 is a distinct hallmark of cancer cells. The median DNA index is shown as a horizontal line in each group.

First, we calculated the DNA index. In 22 women, the samples were aneuploid. Of these, 20 were hyperploid, with a DNA index from 1.1 to 1.7 and a median 1.24, while 1 was hypoploid with a DNA index of 0.9. The other 20 samples were diploid. The results following DNA index calculation are presented in Table S1. The results of ploidy assessment are presented in Figure 2 and Table S2.

**Figure 3:**
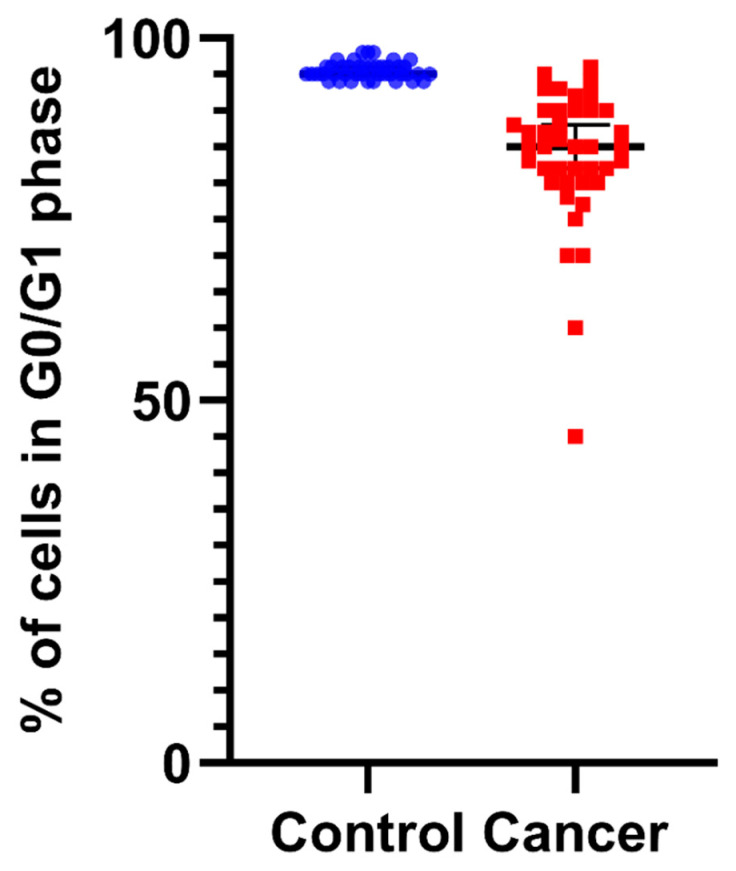
The percentage of cells in G0/G1 in normal control samples versus tumor cells, as quantified by iFC: Percentages of cells in G0/G1 are presented as blue or red dots—for normal and cancer tissue, respectively—in individual patient samples. Median G0/G1 percentages are shown as horizontal lines in each group. There is a significant difference between the two distributions, with cancers cells exhibiting a significantly lower percentage in the G0/G1 cell-cycle phase.

Next, we analyzed the distribution of cells in the various phases of the cell cycle. The mean G0/G1 fractions as values for normal and cancer tissue samples were 95.5 ± 1.13% and 83.8 ± 9.5%, respectively (Figure 3 and Table S2). The G0/G1 cell-cycle phase in normal cells was characterized as significantly higher (*p* < 0.001) than that in cancer cells (Table S3).

**Figure 4:**
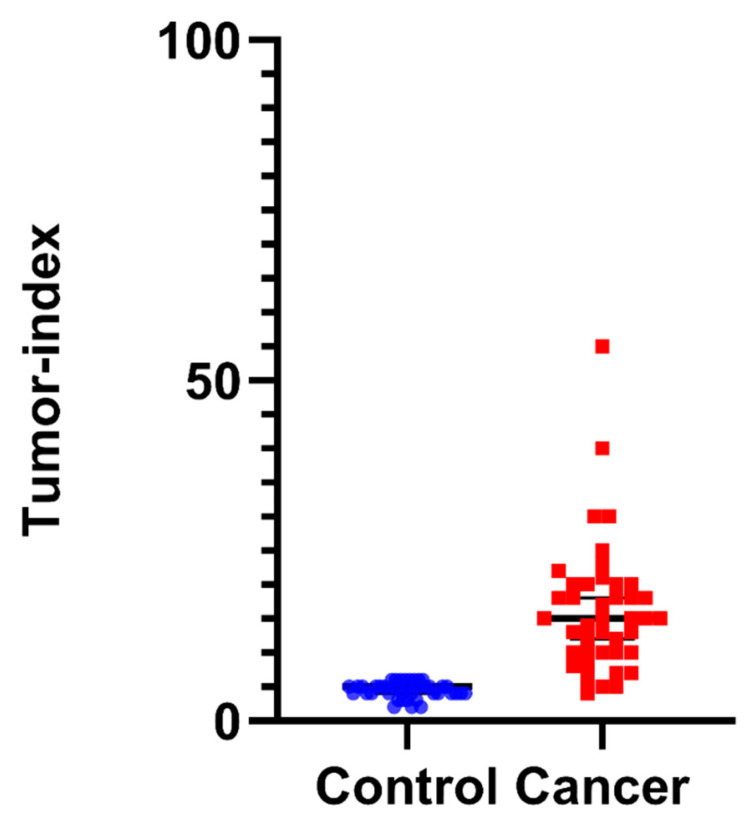
Quantification of tumor index in normal control samples versus tumor cells, as quantified by iFC: Tumor index scores (the sum of the % of cells in the S and G2/M cell-cycle phases) of individual patient samples are presented as blue or red dots, for normal and cancer tissue, respectively. Median percentages are shown as horizontal lines in each group. There is a significant difference between the two distributions, with cancer cells exhibiting a higher tumor index, indicative of malignancy.

On the other hand, the tumor index was significantly increased from 4.5 ± 1.13% to 16.31% ± 9.60%. The highest tumor index was calculated at 55%, while the median was 15% (Table S2). Collectively, the proliferative potential of cancer cells is mirrored in the increase in the tumor index, as can be seen in Figure 4.

**Figure 5:**
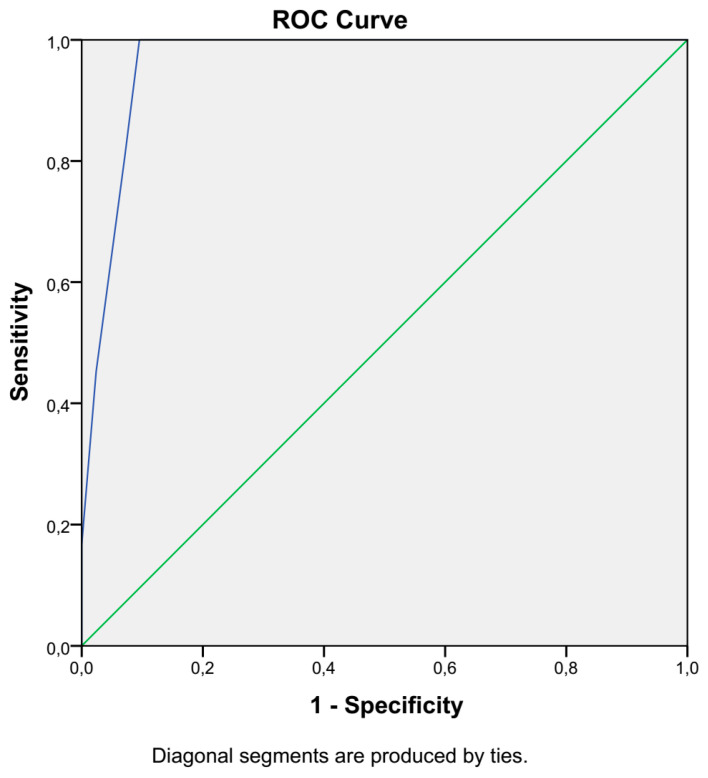
iFC evaluation of gynecological neoplasia is highly sensitive and specific. The results of ROC curve analysis are presented. The horizontal axis represents 1-specificity, and the vertical axis represents sensitivity. The blue line represents the accuracy values, based on both values.

Receiver operating characteristic (ROC) curve analysis was used to evaluate both the sensitivity and specificity of the technique, as well as to estimate the ideal cutoff value for the discrimination of normal tissue from cancer tissue (Figure 5). The optimal cutoff value to delineate the status of tumor tissue is a tumor index of more than 6.5% (or a G0/G1 percentage of less than 94.5%), resulting in 100% sensitivity and 90.5% specificity (Table S4), thus elevating the accuracy of our assay to 95.25%.

## 4. Discussion

Flow cytometry is a powerful tool for detailed and rapid analysis of heterogeneous cell populations, increasing sensitivity to the single-cell level. This method offers numerous advantages, and can be successfully used for tumor diagnosis, as clonal expansion of cancer cells can be monitored as subpopulations with distinct genetic characteristics. It contributes towards precise intraoperative identification of tumor margins, offering the potential of complete removal, and is currently applied in brain [21,22], head-and-neck [23,24], breast [25,26,27], liver [28], pancreatic [29], and colorectal neoplasms [30]. To the best of our knowledge, the present study is the first application of iFC in the detection and characterization of cancer cells in gynecological malignancies.

The hallmarks of cancer include genomic instability and induction of mutations, as well as evasion of growth suppressors and induced and sustained proliferative signaling [39,40]. Chromosomal instability is a prevalent feature of the majority of human cancers, since cancerous cells are frequently characterized by an increased rate of changes in chromosome quantity and structure [41]. Thus, genomic instability is associated with chromosomal abnormalities [42,43], To this end, aneuploidic phenomena can be monitored intraoperatively as changes in DNA content with the aid of iFC, measuring the relative PI fluorescence intensity—as mirrored by DNA index alterations—of cancer cells compared to their normal counterparts (Figure 2).

Furthermore, cancer cells can avoid apoptosis cascade by retaining proliferative capacity and evading growth suppression signaling [40]. This proliferative signaling [44], linked with evasion of growth suppressors [45], can aid in the detection of cancer, since a larger percentage of cells will cycle in the S and G2/M phases. Consequently, the disturbance of cell-cycle distribution in different phases of the cell cycle explains the significant accumulation of cells in the S/G2 phases that can be directly extracted by the tumor index variable, which is associated with an increased proliferative potential (Figure 4). In parallel, the fact that the percentage of G0/G1 cells is significantly lower in all cancer samples when compared to controls is independent proof of increased proliferative potential—a hallmark of malignancy (Figure 3).

Regarding endometrial cancer, intraoperative flow cytometry analyses are of crucial importance since, during surgery, they can quickly (i.e., within a few minutes) provide information regarding the depth of myometrial invasion, as well as whether to proceed with lymph node dissection. Therefore, surgeons can obtain immediate answers as to whether the section removed corresponds to healthy or malignant tissue, enabling them to convert their surgical plans for the benefit of the oncological patient. Along with patients’ age, histological features, and FIGO classification, the percentage of myometrial invasion can be an additional parameter in predicting the risk of recurrence or remission and the probability of disease-free survival [46]. The aforementioned information is of key importance in chemotherapy or radiation planning. Proper tumor excision can minimize the probability of cancer cells’ dissemination and the risk of future secondary malignancies.

The present manuscript represents a single-center study. This constitutes a limitation, since our results should be verified in larger multicenter studies, in which the potential of iFC to characterize tumor cells could be verified with a larger number of samples from different types of gynecological malignancies. Another limitation is the fact that flow cytometric DNA investigation is a single-parameter analysis. Even though we have proven the accuracy of iFC method based on DNA content quantification in several types of cancer, a more accurate cancer cell characterization could be acquired by immunophenotypic flow cytometric analysis, as we have previously have seen in glioma [47,48]. The development of such analysis, which includes immunophenotyping beyond DNA analysis, may offer novel insights into the diagnostic, prognostic, and therapeutic value of iFC.

It is a fact that, when addressing imaging studies, MRI provides the highest level of accuracy in the assessment of tumor size and location (approximately 93 and 91%, respectively, in cervical tumors). The ability to estimate tumor boundaries and local tumor extension is elevated using flow cytometry as, according to the ROC curve, the best sensitivity and specificity cutoff values were established (100% sensitivity and 90.5% specificity, elevating the accuracy of our method to 95.25%, which is above that provided by MRI). The present study, including 42 women, showed total accordance between flow cytometry and pathology evaluation [49].

In conclusion, based on the results so far, DNA content and cell-cycle analysis provide reliable information for the identification of neoplastic lesions and for the assessment of tumor aggressiveness. Thus, this method could be applied as an adjunct to the standard histopathological evaluation of tumor samples in the field of gynecological malignancies, so as to provide a rapid and accurate characterization of malignancy during surgery. The high sensitivity and specificity found in our first, single-center study warrants the further investigation of our methodology in larger, multicenter studies. Based on our results, we suggest that the application of iFC has the potential to represent a major achievement in the field of surgical oncology.

## Data Availability

The data presented in this study are available upon reasonable request to the corresponding author.

## Supplementary Materials

The following supporting information can be downloaded at: https://www.mdpi.com/article/10.3390/biology11091339/s1, Table S1: Patients characteristics. Table S2: Descriptive statistics for G0/G1, Tumor index and DNA-index, cell percentage in control vs cancer cell populations. Table S3: Results of Mann-Whitney test between normal and cancer samples for G0/G1. Table S4: ROC Analysis results for discrimination between normal and cancer cells, based on G0/G1 values and determination of optimal cutoff value.
