# Supplementary material for: Intraoperative Flow Cytometry for the Characterization of Gynecological Malignancies
Source: Biology (Basel). 2022 Sep 11;11(9):1339. doi: 10.3390/biology11091339 (PMC9495699; doi:10.3390/biology11091339)
Supplement: Supplementary file 1 [file biology-11-01339-s001.zip › biology-1870882-supplementary.pdf]

Table S1: Patients characteristics

Patient number	AGE	G0/G1	S	G2/M	DNA index	Tumor Index	Pathology Report
**1**	41	95	2	3	1	5	complex endometrial hyperplasia with atypia
**2**	81	82	5	13	1,5	18	Ovarian Cancer
**3**	72	75	10	15	1,2	25	Ovarian Cancer
**4**	52	90	7	3	1	10	Endometrial Cancer
**5**	52	94	4	2	1	6	Endometrial Cancer
**6**	80	80	13	7	1	20	Endometrial Cancer
**7**	77	88	5	7	1	12	Endometrial Cancer
**8**	62	85	5	10	0,9	15	Ovarian Cancer
**9**	51	85	7	8	1	15	Endometrial Cancer
**10**	35	80	7	13	1	20	cervical cancer
**11**	73	45	17	38	1	55	Endometrial Cancer
**12**	69	87	4	9	1	13	Endometrial Cancer
**13**	72	78	15	7	1,1	22	Endometrial Cancer
**14**	63	87	3	10	1	13	Ovarian Cancer
**15**	56	93	2	5	1,1	7	Endometrial Cancer
**16**	59	70	10	20	1,4	30	Ovarian Cancer
**17**	81	70	15	15	1,7	30	Endometrial Cancer
**18**	59	86	4	10	1	14	Ovarian Cancer
**19**	72	82	8	10	1,6	18	Uterine Sarcoma
**20**	65	96	1	3	1,3	4	complex endometrial hyperplasia with atypia
**21**	70	90	3	7	1,1	10	Endometrial Cancer
**22**	62	60	15	25	1	40	Endometrial Cancer
**23**	58	80	10	10	1	20	Endometrial Cancer
**24**	74	90	5	5	1	10	Endometrial Cancer
**25**	60	92	4	4	1,4	8	Endometrial Cancer
**26**	57	90	4	6	1,1	10	Endometrial Cancer
**27**	74	87	12	1	1	13	Endometrial Cancer
**28**	63	83	5	12	1	17	Endometrial Cancer
**29**	44	85	10	5	1,1	15	Endometrial Cancer
**30**	40	85	10	5	1,1	15	Endometrial Cancer
**31**	55	85	5	10	1	15	Endometrial Cancer
**32**	68	77	9	14	1	23	Endometrial Cancer
**33**	71	90	3	7	1,1	10	Endometrial Cancer
**34**	63	88	8	4	1,1	12	Endometrial Cancer
**35**	58	93	4	3	1,2	7	Endometrial Cancer
**36**	76	80	10	10	1,2	20	Endometrial Cancer
**37**	71	82	8	10	1,6	18	Uterine Sarcoma
**38**	64	83	14	7	1,3	21	Endometrial Cancer

**39**	66	82	8	10	1,1	18	Endometrial Cancer
**40**	54	82	8	10	1,1	18	Endometrial Cancer
**41**	61	92	4	4	1	8	Endometrial Cancer
**42**	70	95	2	3	1	5	Endometrial Cancer

Table S2: Descriptive statistics for G0/G1, Tumor index and DNA-index, cell percentage in control vs cancer cell populations.

Descriptives[a]

	Group			Statistic	Std. Error
**G0/G1**	Control	Mean		95,500	,1746
		95% Confidence Interval for Mean	Lower Bound	95,147	
			Upper Bound	95,853	
		5% Trimmed Mean		95,444	
		Median		95,000	
		Variance		1,280	
		Std. Deviation		1,1316	
		Minimum		94,0	
		Maximum		98,0	
		Range		4,0	
		Interquartile Range		1,0	
		Skewness		,583	,365
		Kurtosis		-,139	,717
	**Cancer**	Mean		83,786	1,4783
		95% Confidence Interval for Mean	Lower Bound	80,800	
			Upper Bound	86,771	
		5% Trimmed Mean		84,828	
		Median		85,000	
		Variance		91,782	
		Std. Deviation		9,5803	
		Minimum		45,0	
		Maximum		96,0	
		Range		51,0	
		Interquartile Range		10,0	
		Skewness		-1,999	,365
		Kurtosis		6,026	,717

DNA index	Cancer	Mean		1,126	,0297
		95% Confidence Interval for Mean	Lower Bound	1,066	
			Upper Bound	1,186	
		5% Trimmed Mean		1,107	
		Median		1,050	
		Variance		,037	
		Std. Deviation		,1926	
		Minimum		,9	
		Maximum		1,7	
		Range		,8	
		Interquartile Range		,2	
		Skewness		1,629	,365
		Kurtosis		1,887	,717
Tumor-Index	Control	Mean		4,500	,1746
		95% Confidence Interval for Mean	Lower Bound	4,147	
			Upper Bound	4,853	
		5% Trimmed Mean		4,556	
		Median		5,000	
		Variance		1,280	
		Std. Deviation		1,1316	
		Minimum		2,0	
		Maximum		6,0	
		Range		4,0	
		Interquartile Range		1,0	
		Skewness		-,583	,365
		Kurtosis		-,139	,717
	Cancer	Mean		16,310	1,4826
		95% Confidence Interval for Mean	Lower Bound	13,315	
			Upper Bound	19,304	
		5% Trimmed Mean		15,278	
		Median		15,000	
		Variance		92,316	
		Std. Deviation		9,6081	

	Minimum	4,0	
	Maximum	55,0	
	Range	51,0	
	Interquartile Range	10,0	
	Skewness	1,953	,365
	Kurtosis	5,835	,717

a. DNA index is constant (=1) when Group = Control. Analysis has been omitted.

Table S3: Results of Mann-Whitney test between normal and cancer samples for G0/G1

Ranks

	Group	N	Mean Rank	Sum of Ranks
G0/G1	Control	42	61,98	2603,00
	Cancer	42	23,02	967,00
	Total	84		

Test Statistics[a]

	G0/G1
Mann-Whitney U	64,000
Wilcoxon W	967,000
Z	-7,370
Asymp. Sig. (2-tailed)	,000

a. Grouping Variable: Control/Cancer

Table S4: ROC Analysis results for discrimination between normal and cancer cells, based on G0/G1 values and determination of optimal cutoff value

Coordinates of the Curve

Test Result Variable(s): G0/G1

Positive if Greater Than or Equal To[a]	Sensitivity	1 - Specificity
44,000	1,000	1,000
52,500	1,000	,976
65,000	1,000	,952
72,500	1,000	,905
76,000	1,000	,881
77,500	1,000	,857
79,000	1,000	,833
81,000	1,000	,738
82,500	1,000	,619
84,000	1,000	,571
85,500	1,000	,452
86,500	1,000	,429
87,500	1,000	,357
89,000	1,000	,310
91,000	1,000	,190
92,500	1,000	,143
[b]**93,500**	**1,000**	**,095**
94,500	,810	,071
95,500	,452	,024
96,500	,167	,000
97,500	,071	,000
99,000	,000	,000

a. The smallest cutoff value is the minimum observed test value minus 1, and the largest cutoff value is the maximum observed test value plus 1. All the other cutoff values are the averages of two consecutive ordered observed test values.

b. Optimal/selected cutoff value is denoted in bold letters.
